# Encouraging to Women Doctors

**Published:** 1882-10-20

**Authors:** 


### *ENCOURAGING TO WOMEN DOCTORS.*

It is said that the President of Mexico has ordered a monthly appropriation of $30 to be made to a distinguished lady who has entered the study of medicine.

